# The Impact of COVID-19 School Closure on Child and Adolescent Health: A Rapid Systematic Review

**DOI:** 10.3390/children8050415

**Published:** 2021-05-19

**Authors:** Sonia Chaabane, Sathyanarayanan Doraiswamy, Karima Chaabna, Ravinder Mamtani, Sohaila Cheema

**Affiliations:** Institute for Population Health, Weill Cornell Medicine—Qatar Education City, Qatar Foundation, P.O. Box 24144, Doha, Qatar; sonia.chaabane.phd@gmail.com (S.C.); sdo4003@qatar-med.cornell.edu (S.D.); kac2047@qatar-med.cornell.edu (K.C.); ram2026@qatar-med.cornell.edu (R.M.)

**Keywords:** school closure, rapid systematic review, COVID-19, child and adolescent health

## Abstract

School closures during pandemics raise important concerns for children and adolescents. Our aim is synthesizing available data on the impact of school closure during the coronavirus disease 2019 (COVID-19) pandemic on child and adolescent health globally. We conducted a rapid systematic review by searching PubMed, Embase, and Google Scholar for any study published between January and September 2020. We included a total of ten primary studies. COVID-19-related school closure was associated with a significant decline in the number of hospital admissions and pediatric emergency department visits. However, a number of children and adolescents lost access to school-based healthcare services, special services for children with disabilities, and nutrition programs. A greater risk of widening educational disparities due to lack of support and resources for remote learning were also reported among poorer families and children with disabilities. School closure also contributed to increased anxiety and loneliness in young people and child stress, sadness, frustration, indiscipline, and hyperactivity. The longer the duration of school closure and reduction of daily physical activity, the higher was the predicted increase of Body Mass Index and childhood obesity prevalence. There is a need to identify children and adolescents at higher risk of learning and mental health impairments and support them during school closures.

## 1. Introduction

The coronavirus disease 2019 (COVID-19) pandemic has affected primary and secondary schooling worldwide. Temporary closure of over 90% of schools worldwide has been reported since March 2020 to mitigate the spread of COVID-19 [1]. This has impacted over 1.5 billion students globally [1]. School closures are driven by physical distancing policies derived from previous models of influenza outbreaks in which children are a vulnerable group for morbidity and play a major role in the spread of the infection [2,3,4]. However, available data for COVID-19 indicates that children (less than 18 years of age) and adolescents (10–19 years) are less susceptible to it than older adults [5], do not appear to significantly drive transmission [6], are a small fraction of the total COVID-19 cases, and have reduced vulnerability to complications as compared to adults [7,8].

In addition to providing knowledge and skills, schools offer an appropriate environment to promote healthy functioning and well-being among children and adolescents; they provide an ideal setting for students to acquire social and emotional skills, as well as behaviors that translate into positive real-life health outcomes [9,10]. Additionally, schools are considered an essential setting for children’s physical activity [11,12]. For children and adolescents with special educational or mental health needs, schools are critical, and in some cases, the only provider of resources that they depend on [13]. School routines also serve as important coping mechanisms for young people with mental health issues [14]. Furthermore, in some middle-income countries, such as Thailand, approximately 60% of all school students below secondary level benefit from the national school feeding programs [15].

With physical school closure, the shift to remote learning became the new norm of education in many countries worldwide. However, not all students have access to online platforms nor the resources required for an optimal learning experience [1]. Moreover, remote learning is challenging and requires the involvement of parents, teachers, schools, and school administrators in the learning process over several months. School closure during the summer months is linked to several negative consequences among children including increased screen time, unhealthy weight gain, and an increase in the prevalence of overweight and obesity [16,17,18]. These negative consequences are also likely to occur during the extended COVID-19 school closures.

School closures associated with the pandemic raise important and urgent questions on how this may affect child and adolescent education, their social life, and health. Previously published literature suggests that epidemics potentially cause emotional harm in both children and their parents, which begins with the concerns about children being infected while attending school [19]. Currently, there is an ongoing debate about whether the advantages of the pandemic-associated school closure and distance learning exceed disadvantages to the child and adolescent physical and psychological well-being, as well as learning and education; and other indirect disadvantages, such as on parents’ mental health and healthcare workforce [20,21,22,23,24,25,26,27]. As the pandemic progresses, newly published studies and reports on the impact of school and childcare closures on child and adolescent health are becoming available. In the absence of clear evidence, the unknown impact of an extended COVID-19-related school closure has concerned parents and policymakers. In such a situation where the evidence is urgently needed to guide decision-making during the current pandemic, carrying out a rapid systematic review has emerged as a useful approach to provide actionable and relevant evidence in a timely and cost-effective manner compared with a standard systematic review [28]. We conducted a rapid systematic review of the literature to synthesize available data on the impact of school closure during the COVID-19 pandemic on child and adolescent health.

## 2. Materials and Methods

We conducted a rapid systematic review following the Cochrane guideline for rapid reviews [29]. The protocol was registered in the Open Science Framework and is available at https://osf.io/n294h (accessed on 16 September 2020). In this review, we synthesize available data on the impact of school closure during the COVID-19 pandemic on child and adolescent health.

### 2.1. Search Strategy and Selection Criteria

A broad search strategy was developed to systematically identify studies on the impact of school closure during the ongoing COVID-19 pandemic on child and adolescent health using keywords and controlled vocabulary. Search terms related to the school/kindergarten/nursery closure and COVID-19 were used. We systematically searched PubMed, Embase, and Google Scholar for grey and non-grey literature between 1 January and 2 September 2020. No restrictions to a specific health condition or language of publication were applied at this stage.

Guidelines for conducting rapid systematic reviews involves a search with at least two literature search strategies limited to the English language [29]. For a comprehensive search, we included three search strategies (for the three databases respectively) and did not apply any language restrictions. For searching primary studies in rapid systematic reviews, it is particularly recommended to search PubMed and Embase databases [29]. It has been suggested that searching PubMed alone, as a proxy to Medline provides sufficient coverage for reviews [30]. Embase serves as a complement to PubMed and is known to produce unique references along with coverage of European and Asian journals [31]. Some evidence has shown that Google Scholar searches often identify different articles than PubMed searches and that Google Scholar articles were more likely to be classified as relevant [32].

We built the search strategy step-by-step following the Cochrane guideline for rapid review. Firstly, a limited search was conducted on PubMed and Embase for relevant articles. The initial search was followed by an analysis of the text words contained in the title and abstract of retrieved articles and of the index terms used to describe the articles. This helped us develop the three concepts for our search strategy: (a) COVID-19, (b) school closure, and (c) adolescent/child health. These concepts and the final choice of our databases were discussed and agreed upon in consultation with senior authors. Thereafter, a set of key terms was developed for our search strategy through a systematic process involving all authors. Because of the variability in the definition of a “child” and an “adolescent” in the published literature, the concept (c) was not combined with other concepts in our search strategy to avoid omitting relevant articles. It is to note that PubMed (MeSH) and Embase (EMTREE) use specific systems of classification of control vocabulary. Our search included words borrowed from the respective systems of the classification (MeSH and EMTREE) used by these databases. However, the Google scholar database permits the use of keywords only, and our search strategy in this database was drawn from our search strategy in PubMed and Embase. Google scholar citations were sorted by date of publication and relevance. Additionally, a hand search of the references from the included studies was also conducted. The detailed search strategy is available in the Supplementary Materials.

### 2.2. Inclusion and Exclusion Criteria

We included all types of studies reporting the impact of COVID-19 school closure on child and adolescent health. We considered school closure when reported in isolation as well as combined with other preventive measures, such as physical distancing, lockdown, and restriction on mass gatherings. Studies published in Arabic, English, French, and/or Urdu (languages spoken by the authors) were eligible to be included. We included quantitative and qualitative primary data on children and adolescents (attending kindergarten, primary, middle, and high schools). We excluded reviews, studies in university settings, and studies examining the indirect impact of school closure such as the impact on COVID-19 transmission, incidence, or mortality, and impact on the parents’ health.

### 2.3. Data Screening

Title, abstract, and full-text screening were conducted by one reviewer and checked by another. Any discrepancies were resolved in consultation with a third reviewer. The retrieved articles were downloaded into Endnote (version X8.2), and duplicates were removed. Discrepancies in the inclusion of primary studies were resolved through discussion with a third reviewer.

### 2.4. Data Extraction

One reviewer extracted the data and another reviewer checked for accuracy. From each included study, the following characteristics were extracted: country, data source, study design, population characteristics, sample size, type and duration of school closure separately or in tandem with other preventive measures, studied outcomes, and results.

### 2.5. Methodological Quality Assessment

To examine the type of evidence, we used the GRADE (grading of recommendations assessment, development, and evaluation) approach. In this approach, randomized clinical trials were considered high-level evidence (level 1), observational studies (such as cohort and case control studies) low-level evidence (level 2), and any other evidence very low-level evidence (level 3) [33,34].

### 2.6. Data Synthesis

For data synthesis and discussion, data related to child and adolescent health were categorized as mental or physical health-related issues. Other findings related to learning, school-based healthcare services, nutrition programs, and dedicated services for special groups were categorized as accessibility issues.

## 3. Results

**Figure 1:**
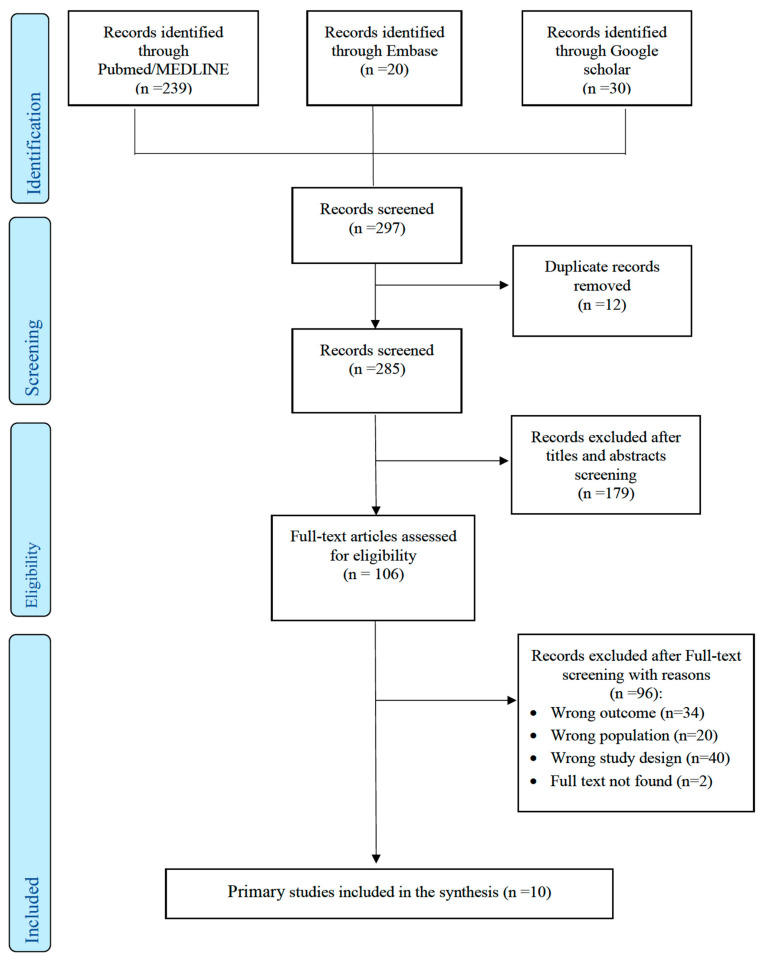
PRISMA 2009 flowchart of the systematic review’s inclusion process.

Our search strategy identified a total of ten studies: nine epidemiological primary studies reporting the impact of COVID-19 school closure on child and adolescent health, and one modeling study that predicted the impact of COVID-19 school closure on child and adolescent health (Figure 1).

Table 1 describes the characteristics of the included primary studies on the impact of school closure on child and adolescent health. These studies were conducted in the USA, Japan, France, Italy, Thailand, and Turkey. In the included studies, the duration of school closure ranged from one week to five months. Seven out of the ten included primary studies reported data on the impact of school closure only on child and adolescent health, whereas the remaining three primary studies reported the impact of school closure combined with other preventive measures (lockdown and home-quarantine) on child and adolescent health.

### 3.1. The Impact on Child and Adolescent Mental Health

School closure and home-quarantine during the pandemic were identified as causes of anxiety and loneliness among the young with a negative effect on children’s behavior (e.g., sleep timing and quality) and psychological well-being (e.g., emotion regulation and self-regulation capacity) with some variation according to the mothers’ working status [35]. The lockdown seems to affect children’s sleep timing rather than their sleep quality [35]. Difficulties to follow a regular routine together with the above changes led to a shift in their daily routines [35]. An increased level of emotional symptoms (e.g., sadness and frustration) among children was reported. Mothers also reported an increase in their children indiscipline and hyperactivity, with a worsening capacity for inhibitory self-control (ability to regulate one’s emotions, thoughts, and behavior) compared to the period prior to school closure [35]. The emotional fatigue in mothers was linked to the disturbance of children’s inhibitory self-control capacity [35]. A study from Florida (USA) reported a 27% decrease in the number of allegations related to child maltreatment in the first two months following school closure [36]. Child and adolescent suicide rates during the first wave of the COVID-19-related school closure were not significantly affected [37].

### 3.2. The Impact on Child and Adolescent Physical Health

A study assessing school closure in combination with lockdown measures reported a significant decrease of 45% in the number of hospital admissions and 68% in the pediatric emergency department (PED) visits for gastroenteritis, bronchiolitis, common cold, and acute otitis media [39].

The only modeling study [38] included in our review was conducted in the USA and predicted an increase in the Body Mass Index (BMI) z-scores and childhood obesity prevalence because of school closure. The longer the duration of school closure and reduction in daily physical activity, higher was the expected increase of the BMI z-scores. The effect of COVID-19-related school closure on obesity among children was modestly larger for boys, non-Hispanic blacks, and Hispanics, as compared to girls, non-Hispanic whites, and Asians.

### 3.3. The Impact on Child and Adolescent Accessibility Issues Related to Learning, Healthcare Services, Nutrition, and Dedicated Services for Special Groups

During school closure, children and adolescents lost access to critical resources for their health and well-being that were normally provided by the school. These included access to (1) school-based healthcare services [41], (2) school and childcare center [15,41] based nutrition programs catering to children from poorer households, and (3) critical resources for children with disabilities, including engagement with specialized educators and structured learning environments [41].

Retrieved qualitative data identified that remote learning presents a challenge for all families and those in poorer households are at a greater disadvantage (lack of support due to parents’ limited availability or resources, lack of access to reliable internet, digital disparities, and lack of access to computer technology), and thus, at increased risk of falling further behind in school due to widening educational disparities [40,41,43].

### 3.4. Quality Assessment

All included studies were observational and hence considered as low-level evidence.

## 4. Discussion

This rapid systematic review identified a total of ten studies reporting both positive and negative impact of the COVID-19 school closure on child and adolescent mental and physical health as well as on accessibility issues related to learning, school-based healthcare services, nutrition, and dedicated services for special groups.

Our results suggest that the COVID-19 school closure made several critical services inaccessible for children and adolescents—school-based healthcare services, essential resources programs for children with disabilities (engagement with specialized educators and structured learning environments), and school and childcare-based nutrition programs providing food to underprivileged children. A positive impact of COVID-19 school closure on child and adolescent health includes a significant decline in the number of hospital admissions and pediatric emergency department visits. Though there was a decline in the number of maltreatment reports for children, it is unclear if this is due to a reduction in the maltreatment incidence or due to decreased reporting. COVID-19-related school closure negatively impacted child and adolescent mental (e.g., anxiety, loneliness, sadness, frustration) and physical (e.g., increased BMI) health morbidity.

A greater risk of widening educational disparities due to the lack of support caused by the limited availability of parents and resources for remote learning was reported among poorer families and among children with disabilities. School closure also contributed to increased anxiety among children and loneliness in young people along with a significant increase in child stress, sadness, frustration, indiscipline, and hyperactivity. Although children’s sleep timings were greatly altered during the lockdown, their sleep quality was, in general, not impacted. An alteration or breakdown in daily routines for youth was also reported. Suicide rates among children and adolescents during COVID-19-related school closure was not affected. The longer the duration of school closure and daily reduction in physical activity, the higher was the predicted increase of the BMI and childhood obesity prevalence.

The duration of school closure was not clear in all the studies. Moreover, none of the included studies linked the duration of school closure with its consequences. However, it can be expected that there would be a greater potential for detrimental ramifications particularly for children living in marginalized communities or poorer households and exposed to interruption of education and malnutrition. In some middle-income countries, such as Thailand, feeding programs provided at schools deliver 30–50% of a child’s nutritional needs daily through the provision of healthier meals. For a considerable proportion of children, one out of the two consumed daily meals is provided at school. These meals are considered a high value for children’s growth and development. School closure for 43 days was expected to lead to almost 270 million missed meals in Thailand. This may lead to a 1–2 kg weight loss—close to 5–10% of a child’s total body weight [15].

Although a limited number of studies are available reporting health consequences of the COVID-19 school closure, the psychological aspect seems to be the standout issue confronting child and adolescent health. Previously, large-scale disasters, whether traumatic, natural, or environmental, were linked to an increase in depression, substance use disorder, and a broad range of other mental and behavioral disorders in children and adolescents [44,45]. Moreover, a study showed that parents’ personal distress levels and child rearing stress scores during the school closures were significantly higher than before school closures [46]. Although psychological disruption is a multifactorial issue, the identification of groups that may be at higher risk can help us support these individuals with the provision of various social connections, including those provided by healthcare professionals, families, and schools [42].

Schools play an active role in promoting health-conscious behavior among children and adolescents. The COVID-19-related school closure and lockdown for several months resulted in children and adolescents restricting their movement that may have led to an increase in physical inactivity and sedentary behavior contributed by an increase in daily screen time (video games, time spent on internet, television, mobile phones) [26]. Suspected short- and long-term consequences of these behavioral changes, ranging from overweight to mental health issues, need further investigation.

While none of the included studies assessed the learning that may have been lost during school closure and distance learning, several concerns related to learning modalities were expressed by schools, teachers, and learners. In small, low-density, and rural school areas in the USA, students were sent home without supplemental education material or clear plans for transitioning to distance learning [43]. A study reported a loss of 1.8 months of progress in mathematical skills and four months of spelling skills during summer school holidays under normal circumstances among students of all socioeconomic status [47]. However, students of low socioeconomic status faced the greatest learning deficit when it came to reading comprehensiveness [47]. Hence, the loss of learning and effect on child and adolescent cognitive development during school closure and distance learning must be researched further to develop a better understanding of this issue.

The advantage of COVID-19 school closure has been questioned globally—some studies show no evidence that school closure may influence the transmission of COVID-19 [48] and interrupt transmission by itself; however, there is some indication that peak incidence of the infection can be reduced [5]. Therefore, policymakers and advisers must take into consideration the effects of school closure on accessibility to critical school related resources (learning, school-based healthcare services, nutrition programs, and dedicated services for special groups), and the physical and mental health of children and adolescents. The outcomes related to health and accessibility issues should be balanced, keeping in view the emerging evidence reporting its weak impact on the interruption of transmission.

To our knowledge, this is the most comprehensive review that synthesizes available data focusing on the impact of COVID-19 school closure on child and adolescent health. The rapid review follows the Cochrane guidelines. Compared with the methods of a systematic review, rapid reviews apply an abbreviated methodology to accelerate the process of producing evidence for stakeholders in a resource efficient manner [29]. Although only one literature source is usually utilized in rapid reviews, we searched three literature sources including grey literature minimizing the chance of missing studies that are relevant to our topic. Since a qualitative synthesis with no clinical implications is urgently needed, results from a rapid review are usually similar to those obtained following a conventional systematic review [49].

An independent dual title and abstract and full text screening might have led to additional relevant studies [50]. Though, we limited our inclusion criteria to specific languages (Arabic, English, French, and/or Urdu), our initial search did not yield publications in languages of countries that were heavily affected by COVID-19, such as, Chinese, Italian, or Spanish. Since COVID-19 continues to evolve rapidly, we may have missed recent publications since our last search was conducted. The evidence provided in this review is limited by the quality of the included observational studies. Given the limited number of published studies on various outcomes (mental and physical health, and accessibility issues), the certainty to which our review findings apply to communities across the board remains unknown. Nevertheless, we are confident that none of the methodological limitations would change the overall conclusions of this review. Larger studies designed to assess the association of school closure with adverse health outcomes in children and adolescents are needed to confirm our findings.

## 5. Conclusions

Findings from our rapid review suggests that the impact of school closures during the COVID-19 pandemic includes loss of access to school-based and critical services and resources particularly for children with disabilities and those living in poorer families. COVID-19 school closures were also associated with increased stress among children and emotional reactions (e.g., sadness, frustration, indiscipline) in addition to the breakdown in daily routines. The longer the duration of school closure and the daily reduction in physical activity, the higher the predicted increase of the BMI and childhood obesity prevalence. Concerns about children and adolescents’ ability to learn during pandemic related school closures needs further follow-up and special consideration in future research and evaluation studies. Harms of school closure on child and adolescent health seem to exceed some indicators of positive health care system effects. It would be helpful to assess other health and social effects—such as the quality of life of children and their families, lifestyle, screen time, education/learning, cognitive development, social interactions including social media use—resulting from school closures. Custom tailored benefit and risk assessments specific to the local socioeconomic context, health system, and school resources are essential when considering school closures.

## Figures and Tables

**Table 1:** Characteristics of the included primary studies on the impact of school closure on child and adolescent health.

Publication Country Data Source	Study Design/Analysis	Population Characteristics	Types of Interventions Duration of School Closure	Outcomes	Findings
An, R., 2020 [38] USA Early Childhood Longitudinal Study	Microsimulation model	Children in kindergarten class of 2010–2011 (*n* = 15,631) were followed from kindergarten through 5th grade 6–10 years	Scenario 1: 2-month nationwide school closure (April and May 2020) with COVID-19 vs. 2-months nationwide school closure in April and May 2020 without COVID-19	Increase in the body mass index z-scores Childhood obesity prevalence	An increase in the mean BMIz by 0.056 unit An increase in childhood obesity prevalence by 0.640 percentage points
			Scenario 2: Scenario 1 + 10% reduction in daily physical activity in the summer from June to August	Increase in the body mass index z-scores Childhood obesity prevalence	An increase in the mean BMIz by 0.084 An increase in childhood obesity prevalence by 0.972 percentage points
			Scenario 3: Scenario 2 + 2-month school closure in September and October; November and December	Increase in the body mass index z-scores Childhood obesity prevalence	An increase in the mean BMIz by 0.141 units An increase in childhood obesity prevalence by 1.676 percentage points
			Scenario 4: Scenario 3 + 2-month school closure in November and December	Increase in the body mass index z-scores Childhood obesity prevalence	An increase in the mean BMIz by 0.198 units An increase in childhood obesity prevalence by 2.373 percentage points
			Comparison of the control scenario without the COVID-19 pandemic and the 4 alternative scenarios with COVID-19	Childhood obesity (BMIz in the 95th percentile or higher in the growth chart)	“Compared to girls and non-Hispanic whites and Asians, the impact of COVID-19 on childhood obesity was modestly larger among boys and non-Hispanic blacks and Hispanics, respectively.”
Angoulvant, F., 2020 [39] France French surveillance data for pediatric emergency department (PED) visits and related hospital admissions	Quasi-experimental time series analysis	871,543 pediatric emergency department (PED) visits	School closure + lockdown 18 March 2020 to 19 April 2020. One week after the start of the lockdown	Number of hospital admissions	“A decrease of −45% in hospital admissions in the period from 1 January 2017 to 17 March 2020 and from 18 March 2020 to 19 April 2020.”
				number of pediatric emergency department (PED) visits for gastroenteritis, bronchiolitis, common cold, acute otitis media	“A decrease of −68% in the overall number of PEDs visits in the period from 1 January 2017 to 17 March 2020 and from 18 March 2020 to 19 April 2020.” “A significant decrease over 70% of acute gastro-enteritis, common cold, bronchiolitis and acute otitis media compared to the expected values. Urinary tract infections were not impacted by the lockdown both regarding overall PEDs visits (−16.4% [−40.8; +6.4]) and hospital admissions (+20.7% [−27.0; +58.5]).”
Ijadi-Maghsoodi, R., 2020 [40] Los Angeles, USA Los Angeles County (LAC) Department of Mental Health (DMH) and its network of community mental health clinics providing critical services to youth and families	Report	Youth and families during the pandemic	School closures in the county	Accessibility to resources	“Teachers expressed concerns about under resourced students facing barriers to accessing virtual learning, heightened student anxiety, and difficulty connecting to students in need, all compounded by a compressed timeline. This includes digital disparities and lack of access to computer technology among families and providers.”
Masonbrink, A.R., 2020 [41] USA National data from diverse sources on Education Nutrition Physical Health Mental Health Child and Family Safety COVID-19 Response Legislation	Pre-publication Release	All children Children in poverty Children with disabilities	School closure	Challenges related to: Educational losses and attainment Accessibility to critical resources Access to the nutrition program Physical and mental health Psychological impact	“Remote learning presents a challenge for all families, those in poverty are at greater disadvantage and thus at increased risk for widening educational disparities.” “Parents in poverty are facing their own pandemic-related stressors (e.g., unemployment, at-risk jobs) and may lack the time or resources to support remote learning.” “Critical access to the nutrition programs that serve 35 million children living in poverty daily, is typically provided through schools and childcare centers.” “School closure means loss of critical resources for children with disabilities, including engagement with specialized educators and structured learning environments.” “Children now face diminished access to healthcare care because of loss of school-based services, increasing parental unemployment, loss of health insurance, and avoidance of health care settings.” “Since the pandemic began, there have been isolated reports of increased child abuse severity, however, numerous states are reporting an ominous decrease in reports to child protective services (CPS), thought to be related to under-recognition.”
Mayurasakorn, K.B., 2020 [15] Thailand National data and observations	Expert-viewpoint	Thai children reside in rural areas Pre-primary and primary school levels	School closure + the national school breakfast and lunch programs	Food and learning insecurity	Schools closure during COVID-19 caused two major threads: When schools close, nutrition is compromised. It is estimated that school feeding programs provide up to 30–50% of a child’s daily nutritional requirements through meals healthier than those prepared at home at the same price. Many children consume only two meals a day and one meal at school is considered high value for children’s growth and development. This would result in almost 270 million missed meals nationwide; for a child who misses school meals this may lead to a 1–2 kg weight loss over the 43 days—close to 5–10% of a child’s total body weight. School closures can bring added financial pressures and risk of child undernutrition for poorer families given the now larger costs of childcare.” “The school closures and childcare restrictions may leave children unattended at home, thereby curtailing children’s learning capacity during school closure. Extended school closures may still lead to widening the learning, health, and nutrition gaps between youth from lower-income and higher-income households. Perhaps worse than educational impediment, an end to the pandemic is yet to arrive, which may lead to grievous economic depression. This can exacerbate existing poverty and as well as children’s nutrition and learning outcomes.”
	Snapshot of strategies/challenges Analysis	Convenience sample of 9 school districts that were closed as part of social distancing strategies Small low-density, rural school district Urban and suburban schools	School closures 16 March and 20 March 2020	Distance learning Nutrition programs	“In a small low-density, rural school district with lower reported instances of COVID-19 at the time of implementation, students were sent home without supplemental education material or clear plans for transitioning to distance learning. In low- to middle-income communities and less affluent suburban counties, school districts provided students with study packets, accessed either online or distributed/picked up at school, given the lack of access to reliable Internet and devices.” “More affluent urban and suburban schools used online instruction platforms. Some used a blend of worksheets, online resources, and online instruction, while schools attempted to provide online-capable devices to students in need. Some broadcasted educational material through public access TV channels, social media, and on their websites, while arranging device distribution. Some were also working with Internet service providers to provide low- or no-cost Internet access. One district employed Wi-Fi-equipped school buses throughout the community for students to access. Meals were provided to students using free or reduced meal programs.”
Kılınçel Ş., 2020 [42] Turkey Facebook family groups, and Google Forms questionnaires sent by the child psychiatry clinic to their smartphones. Sociodemographic form, State-Trait anxiety scale, and UCLA loneliness survey were used as data collection tools.	Cross-sectional	745 adolescents aged between 12 and 18 years 13 different schools	School closure and home-quarantine	Risk of anxiety and loneliness	“Closure of schools and home-quarantine during pandemic causes anxiety and loneliness in young people.”
Isumi A., 2020 [37] Japan Public data on suicide statistics Public data on population estimates	Descriptive study	Children under 20 years	School closure between January 2018 and May 2020	Impact on suicide rates	“The first wave of the COVID-19 pandemic has not significantly affected suicide rates among children and adolescents during the school closure in Japan.”
Baron E.J., 2020 [36] Florida, USA Country and district-level data Florida DCF	Descriptive study	School staffing for each of the 67 counties in Florida	School closure March to April 2020	The number of child maltreatment allegations	“A counterfactual distribution of child maltreatment allegations for March and April 2020, the first two months in which Florida schools closed. The actual number of reported allegations was approximately 15,000 less (27%) than expected for these two months.”
Di Giorgio E., 2020 [35] Italy Subjective Time Questionnaire (STQ) The Behavior Rating Inventory of Executive Functions—preschool version (BRIEF-P) Executive Functions (EF) Self-Control Index (ISCI)	Survey	245 mothers and their pre-school children aged between 2 and 5 years	Closure of schools, individual home confinement, and the related social restrictions from 1st to the 9th of April (after three weeks of confinement) compared to the week before the national lockdown	Mothers and their pre-school children’s behavioral habits (i.e., sleep timing and quality, subjective time experience) and psychological well-being (i.e., emotion regulation, self-regulation capacity).	“Restrictive measures had negative effects on mothers’ and their children’s behavioral and psychological levels, with some differences depending on the mothers working situation.” “Besides their and their mothers’ sleep quality, children’s inhibitory self-control capacity seemed to be also associated with mothers’ trait emotional fatigue. Children’s sleep quality was, on average, less affected by the lockdown, although their sleep timing strongly shifted (they went to bed ~53 min and woke up ~66 min later than usual). These changes, together with reported difficulties of following routines, indicate a substantial breakdown in their daily routines.” “Mothers reported an increasing level of emotional symptoms such as sadness and frustration, whereas they perceived their children as more undisciplined and hyperactive, with a worsening inhibitory self-control capacity.”

Note: COVID-19: the coronavirus disease 2019; UCLA: University of California, Los Angeles; DCF: Department of Children and Families.

## Data Availability

The datasets generated during and/or analysed during the current study are available from the corresponding author on reasonable request.

## Supplementary Materials

The following are available online at https://www.mdpi.com/article/10.3390/children8050415/s1. Table S1. The 2009 PRISMA checklist for reporting a systematic review. Panel 1: Search Strategy.
